# Sponge Tents

**Published:** 1869-07

**Authors:** 


Sponge Tents.—Knowing the fact that absolute or *strong* alcohol will quickly *set* the fibres of common sponge, after having been moulded or compressed into any given size or shape, I was led to the following quick and easy method of preparing sponge tents, tampons, etc :

The sponge is first thoroughly moistened with water and pressed as dry as the strength of the hand will permit; then having formed it into the desired shape and size by the hand, or by pressing into a quill or any other tube or mould it is immersed into the alcohol. If the spirit is sufficiently strong (90 to 100 per cent.) the sponge is *immediately* set into the given shape, which it retains perfectly after the pressure or mould is removed. It is then hard, firm and inflexible and may be trimmed to a sharp point or any other desired shape.

To restore it to its former size and shape it is only necessary to moisten it with a few drops of water. The alcohol sets the sponge perfectly, whether the amount of compression be much or little, so that the degree of dilatation, attainable by the use of tents thus prepared, will of course, depend upon the size after moulding and the degree of pressure used. As this process of preparation works perfectly and without delay its advantages are obvious.

